# Molecular Pathology of Well-Differentiated Pulmonary and Thymic Neuroendocrine Tumors: What Do Pathologists Need to Know?

**DOI:** 10.1007/s12022-021-09668-z

**Published:** 2021-02-27

**Authors:** Marco Volante, Ozgur Mete, Giuseppe Pelosi, Anja C. Roden, Ernst Jan M. Speel, Silvia Uccella

**Affiliations:** 1 Department of Oncology, University of Turin, Turin, Italy; 2 Departments of Pathology, University Healthy Network and University of Toronto, Toronto, Canada; 3 Department of Oncology and Hemato-Oncology, University of Milan, Milan, Italy; 4 Department of Laboratory Medicine and Pathology, Mayo Clinic Rochester, Rochester, MN USA; 5 Department of Pathology, GROW-School for Oncology and Developmental Biology, Maastricht University Medical Center, Maastricht, Netherlands; 6 Dept. of Medicine and Surgery, University of Insubria, Varese, Italy

**Keywords:** Neuroendocrine tumor, Carcinoid, Lung, Thymus, Molecular, Germline variants, MEN1, RAD5A1C, DIPNECH

## Abstract

Thoracic (pulmonary and thymic) neuroendocrine tumors are well-differentiated epithelial neuroendocrine neoplasms that are classified into typical and atypical carcinoid tumors based on mitotic index cut offs and presence or absence of necrosis. This classification scheme is of great prognostic value but designed for surgical specimens, only. Deep molecular characterization of thoracic neuroendocrine tumors highlighted their difference with neuroendocrine carcinomas. Neuroendocrine tumors of the lung are characterized by a low mutational burden, and a high prevalence of mutations in chromatin remodeling and histone modification-related genes, whereas mutations in genes frequently altered in neuroendocrine carcinomas are rare. Molecular profiling divided thymic neuroendocrine tumors into three clusters with distinct clinical outcomes and characterized by a different average of copy number instability. Moreover, integrated histopathological, molecular and clinical evidence supports the existence of a grey zone category between neuroendocrine tumors (carcinoid tumors) and neuroendocrine carcinomas. Indeed, cases with well differentiated morphology but mitotic/Ki-67 indexes close to neuroendocrine carcinomas have been increasingly recognized. These are characterized by specific molecular profiles and have an aggressive clinical behavior. Finally, thoracic neuroendocrine tumors may arise in the background of genetic susceptibility, being MEN1 syndrome the well-defined familial form. However, pathologists should be aware of rarer germline variants that are associated with the concurrence of neuroendocrine tumors of the lung or their precursors (such as DIPNECH) with other neoplasms, including but not limited to breast carcinomas. Therefore, genetic counseling for all young patients with thoracic neuroendocrine neoplasia and/or any patient with pathological evidence of neuroendocrine cell hyperplasia-to-neoplasia progression sequence or multifocal disease should be considered.

## Introduction

Pulmonary neuroendocrine tumors (pulmonary carcinoid tumors) are well-differentiated epithelial neuroendocrine neoplasms that represent about 25% of all neuroendocrine neoplasms [1]. Unlike gastro-entero-pancreatic neuroendocrine tumors, pulmonary neuroendocrine tumors are diagnosed in the vast majority as early-stage disease. Lymph node involvement is present in about 20% of cases [2]. Stage III and stage IV diseases are encountered in about 10% of patients, each [3, 4]. Thymic neuroendocrine tumors represent less than 5% of all thymic and mediastinal neoplasms and 0.4% of all neuroendocrine tumors. In general, thymic neuroendocrine tumors behave more aggressively than pulmonary neuroendocrine tumors, they are frequently metastatic, and 5-year survival is not reached in approximately 50% [5]. Patients with these tumors are typically symptomatic, often secrete ectopic hormones (e.g., ACTH in 40% of tumors) and, like neuroendocrine tumors of the lung, secrete circulating biomarkers such as chromogranin A and 5-HIAA (metabolite of serotonin).

### Histopathological Classification

World Health Organization (WHO) classification of thoracic tumors categorizes neuroendocrine tumors of the lung and thymus as typical (< 2 mitoses per 2 mm^2^ and no necrosis) and atypical (2–10 mitoses per 2 mm^2^ and/or necrosis) [6]. Tumor histology is one of the most powerful predictors of aggressive behavior in lung and thymic carcinoids. In fact, atypical carcinoid histology is an independent variable of adverse prognosis even in lung cases at the metastatic stage [7]. However, this classification scheme has been designed for resected primary tumors to predict the risk of recurrence or metastasis, whereas the clinical impact of these categories in metastatic setting is undetermined. Indeed, mitotic index cut off values per se have been recently called into question in a large series of over 700 well-differentiated lung neuroendocrine tumors [8]. Furthermore, the impact of Ki-67 labeling index as an adjunct in the subclassification of pulmonary carcinoids into distinct prognostic subgroups has been a matter of long debate [9, 10]. Even recently, models that combine histology and proliferation index evaluation have been proposed [11], although not reaching a consensus that allows a definitive clinical application.

### The Grey Zone Between Thoracic Neuroendocrine Tumors and Neuroendocrine Carcinomas

**Fig. 1:**
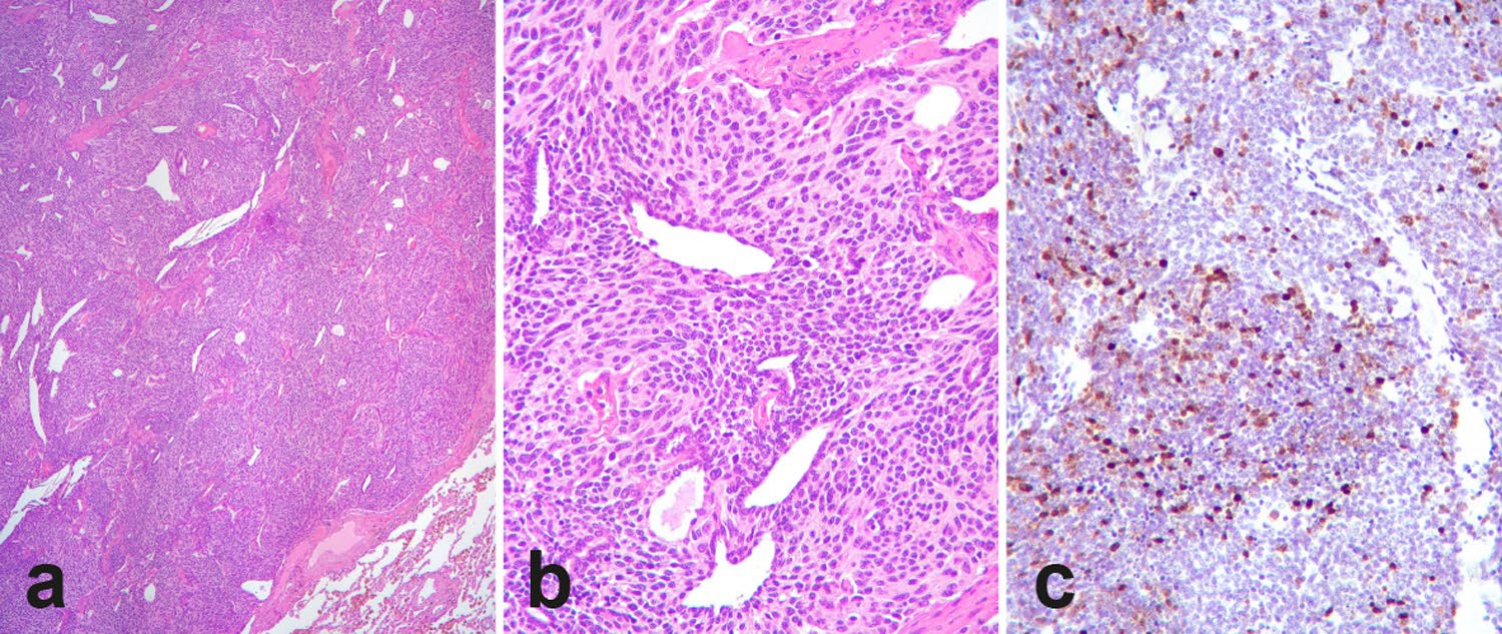
Atypical pulmonary carcinoid with high proliferation index. A well-circumscribed lung lesion (**a**) with spindle cell morphology—well differentiated—with mitotic index of 5 per 2 mm^2^ (**b**) displays heterogeneous “clonal” proliferation, as assessed by means of Ki-67, with up to 40% proliferation index in hot spots (**c**)

As a matter of fact, several pathological and clinical evidences suggest that typical and atypical carcinoid tumors should not be considered as distinct histotypes; they rather represent a unique group of lesions, the prognosis of which is driven by the presence or absence of a set of pathological features of aggressiveness. Moreover, the border between neuroendocrine tumor groups and thoracic neuroendocrine carcinomas (especially with large cell neuroendocrine carcinoma) is not clear cut, and a grey zone where morphology does not fully match with proliferation has been recognized. On the one side, a growing body of studies indicates that a subset of lung neuroendocrine tumors that are classified as pulmonary carcinoids based on mitotic index (therefore not above 10 mitotic figures in 2mm^2^) have a significantly higher Ki-67 proliferation rate than the mean of overall carcinoid tumors [12, 13]. These cases, although maintaining a well differentiated morphology, have Ki-67 proliferation index close to large-cell neuroendocrine carcinomas and display a more aggressive clinical behavior than those with lower proliferative rates, irrespective of the carcinoid histotype assessed by the WHO classification [14, 15] (Fig. 1). On the other side, some lung neuroendocrine neoplasms classified as large-cell neuroendocrine carcinomas based on the high mitotic rate maintain a well-differentiated morphology, with a well-preserved organoid structure, focal necrosis—if present—and inconspicuous nucleoli [16]. These tumors, apart from classification issues, raise also the possibility of a direct progression from neuroendocrine tumors to neuroendocrine carcinomas. Although rarely demonstrable in the clinical practice [17], this possibility has been hypothesized based on clinical and molecular data [18].

**Fig. 2:**
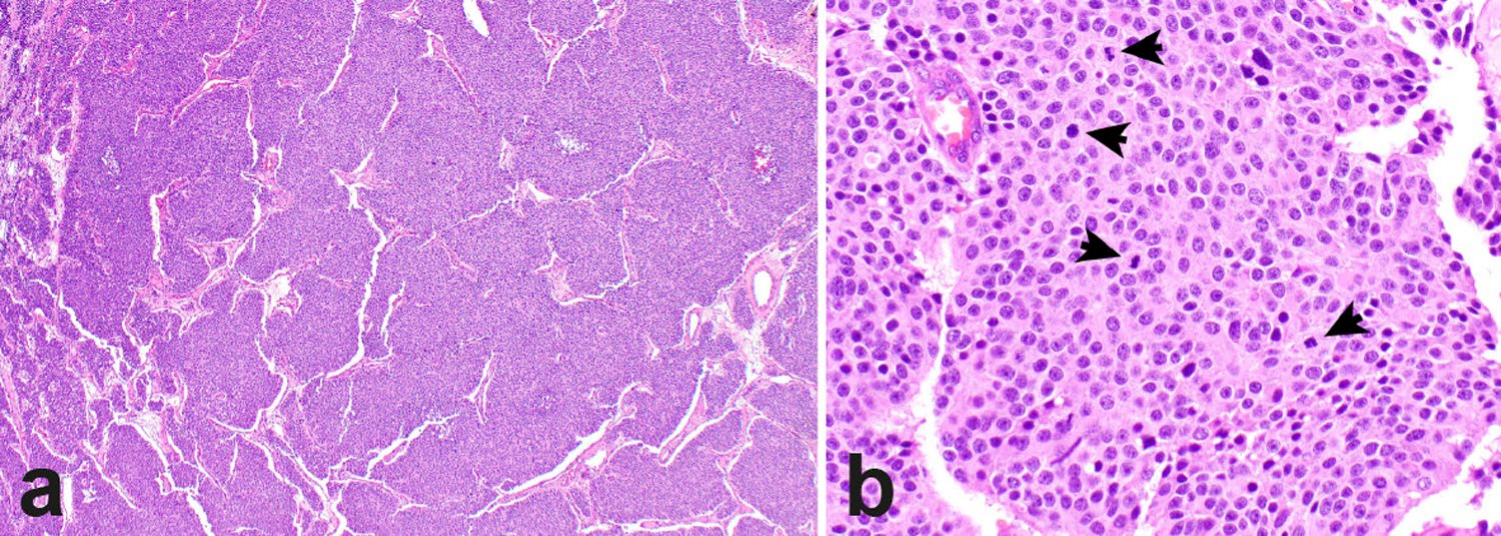
Thymic neuroendocrine tumors with morphologic features of an atypical carcinoid tumor but with increased mitotic activity. The tumor shows a nested growth pattern and lacks large areas of necrosis (**a**). High magnification reveals bland appearing round to oval cells with a fair amount of cytoplasm and nuclei with inconspicuous nucleoli, lacking prominent nucleoli. Mitotic activity is increased (17 mitoses per 2mm^2^, arrows) (**b**)

Similar to neuroendocrine tumors elsewhere, the morphologic classification alone may fail to capture the heterogeneous spectrum of thymic neuroendocrine neoplasms. One of the best examples is the occurrence of thymic neuroendocrine tumors that show morphologic features of an atypical carcinoid but have a mitotic activity that is over 10 mitoses per 2mm^2^ [19, 20] (Fig. 2). Similar to neuroendocrine tumors of the lung, limited evidence suggests that the morphologic classification of thymic neuroendocrine tumors might not accurately predict the outcome of all of these tumors. Specifically, there appears to be overlap between atypical carcinoid tumors and large-cell neuroendocrine carcinomas.

### Molecular Sub-classification of Thoracic Neuroendocrine Neoplasms

**Fig. 3:**
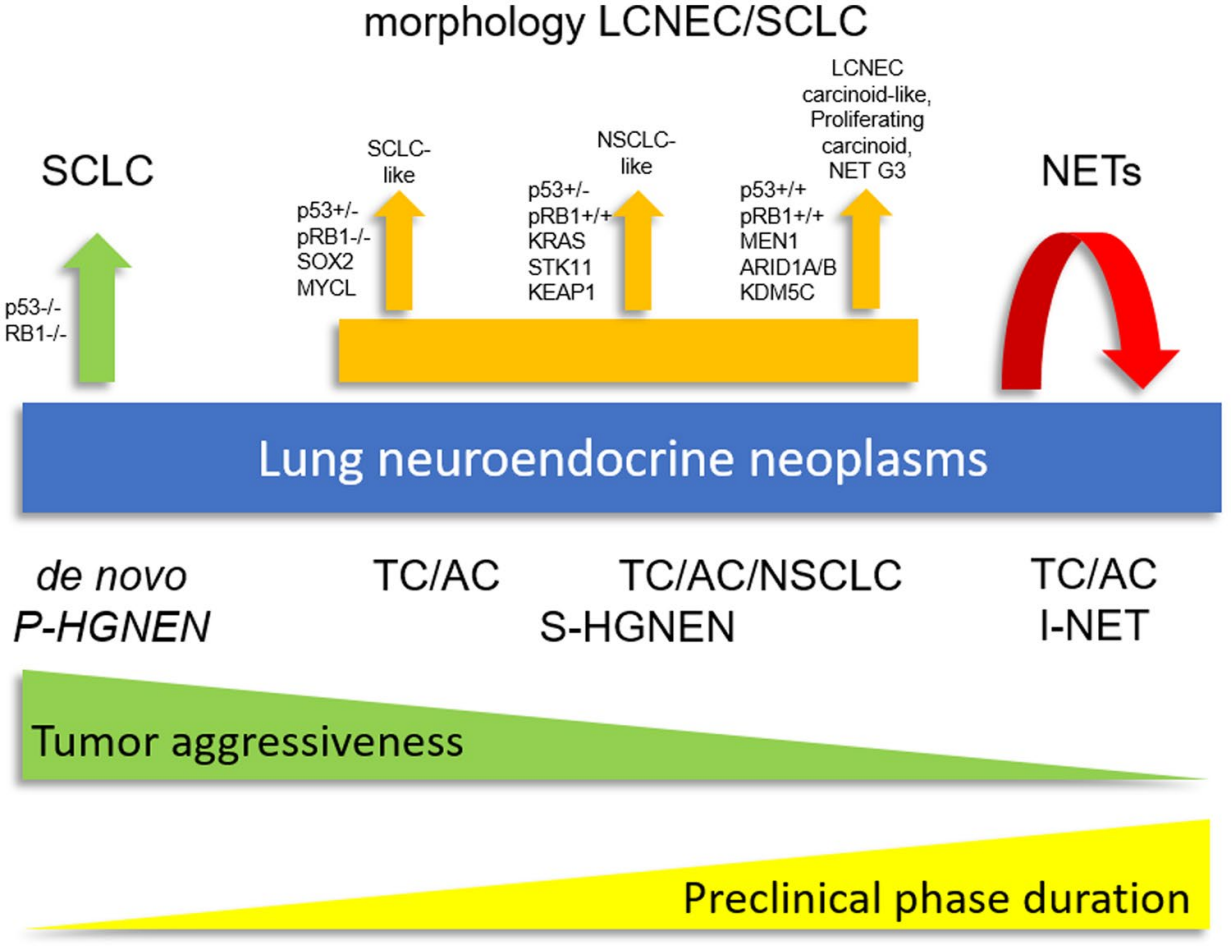
Reappraisal of the lung neuroendocrine neoplasia spectrum according to diverse developmental pathways and putative natural history of disease. Green and yellow triangles with opposite slope indicate tumor aggressiveness and putative duration of preclinical phase, respectively. LCNEC large-cell neuroendocrine carcinoma, SCLC small-cell lung carcinoma, NET neuroendocrine tumor, P-HGNEN primary high grade neuroendocrine neoplasm, S-HGNEN secondary high-grade neuroendocrine neoplasm, I-NET indolent neuroendocrine tumor, TC typical carcinoid, AC atypical carcinoid, NSCLC non-small cell lung cancer

In the last years, a more accurate description of genomic, epigenetic, and gene expression profiles in thoracic neuroendocrine tumors, with special reference to those in the lung, has been achieved. Overall, the available data not only provided prevalence rates of molecular alterations in distinct histological subtypes of neuroendocrine tumors of lung and thymus, but also supported the existence of intermediate molecular classes of thoracic neuroendocrine tumors that parallel the pathological observation of lesions that do not completely fulfill the mitotic index/necrosis-based classification. A descriptive graph illustrating the spectrum of pulmonary neuroendocrine neoplasms according to diverse developmental pathways and putative natural history of disease is reported in Fig. 3. In this scenario, two subsets of neuroendocrine carcinomas are identified [21]. Primary neuroendocrine carcinomas feature undifferentiated tumor cells characterized by severe gene alterations (e.g., biallelic inactivation of *RB1* and *TP53*) responsible for de novo pathogenetic mechanisms. They are characterized by a very short preclinical phase and high clinical aggressiveness with extensive disease at presentation. These tumors account for the vast majority (approximately 70%) of neuroendocrine carcinomas.

In terms of morphologic and molecular characteristics, secondary neuroendocrine carcinomas, which are linked to tumor progression from pre-existing lesions, often show greater heterogeneity than primary ones. Similar tumors occur in both the lung and the thymus [20]. In this setting, secondary neuroendocrine carcinomas can develop through the acquisition of diverse genetic alterations either from neuroendocrine tumors (carcinoids) at high risk of progression or from non-small cell lung carcinomas. Duration of the preclinical phase is inversely related to tumor aggressiveness. Conversely, carcinoid-like large-cell neuroendocrine carcinoma would merge within the recently proposed category of neuroendocrine tumors with elevated proliferation rates in the lung (similar to gastro-entero-pancreatic neuroendocrine tumors with high grade proliferation; Grade 3 NET) [22] and might derive from neuroendocrine tumors (carcinoids) that have an increased risk of progression. These tumors would be characterized by a variably long preclinical phase (especially carcinoid-like large cell neuroendocrine carcinomas) and a clinical aggressiveness that is somewhat intermediate between de novo neuroendocrine carcinomas and indolent neuroendocrine tumors. These tumors would show lower propensity to metastasis formation at presentation. Finally, there are also indolent neuroendocrine tumors that are destined to remain unchanged over time and feature carcinoids with the longest preclinical phase and negligible clinical aggressiveness.

### Genomic Profiling of Pulmonary Neuroendocrine Tumors (Carcinoid Tumors)

The genomic profiling of pulmonary neuroendocrine tumors (carcinoids) has been recently investigated by means of high-throughput next-generation techniques [23]. Despite some heterogeneity of results in terms of prevalence of mutations in individual genes, molecular studies of pulmonary neuroendocrine tumors overall indicate that:

(i) Carcinoids are characterized by a low mutational burden, with a slight increase from typical to atypical carcinoids, but invariably lower than large-cell and small-cell neuroendocrine carcinomas;
(ii) G:C > A:T transitions, which are characteristic of smoking exposure, are less frequent in carcinoids as compared with neuroendocrine carcinomas;
(iii) Chromatin remodeling and histone modification-related genes are mostly altered, whereas mutations in genes frequently altered in neuroendocrine carcinomas (such as *RB1* and *TP53*) are very rare but not absent.

Globally, mutations in chromatin remodeling genes have been detected with variable frequencies, in up to more than 40% of pulmonary neuroendocrine tumors in some studies [24–27]. Lower rates of mutations in chromatin remodeling genes have been detected in other studies [28, 29], probably as the result of differences in variant calling algorithms as well as different filtering parameters, thus emphasizing the need for standardization of bioinformatics and uniformity of quality control [30]. Genes involved in chromatin remodeling are divided in two main groups: those encoding for components of the covalent histone-modifying complexes (acetylation, methylation, phosphorylation, and ubiquitination) and those encoding for complexes of adenosine triphosphate–dependent chromatin remodeling (such as the SWI/SNF). Overall, *MEN1* is the most frequently somatically mutated gene in neuroendocrine tumors (identified in 11–22% of carcinoids) [31, 32], which is usually associated to loss of heterozygosity [23, 24]. *MEN1* is a histone modifier acting as a scaffold protein into a ternary complex composed of *MEN1-MLL-PC4* and *SFRS1* interacting protein 1 gene (*PSIP1*). Interestingly, *PSIP1* has also been found to be mutated in a subset of neuroendocrine tumors (5%) lacking *MEN1* gene alterations [24]. Interestingly, *MEN1* gene mutations and reduced gene expression have been associated with poor prognosis in pulmonary neuroendocrine tumors, thus representing a prognostic molecular biomarker in these neoplasms [25, 33]. Other genes belonging to the chromatin remodeling pathway include those of the KMT2 (MLL) family of covalent histone modifiers whose mutation rate is up to 14% [25]. Genes of the SWI/SNF complex were also found to be mutated in more than 20% of neuroendocrine tumors of the lung, including genes of the ARID1 family, *BCL11A*, *SMARCA1*, *SMARCA2*, *SMARCA4*, *SMARCB1*, and *SMARCC2* [24, 25].

Other pathways are by far less commonly altered in pulmonary neuroendocrine tumors. Recurrent mutations in the kinase domain of *PIK3CA* (exon 9 and 20) have been described in 13% of typical carcinoids and 39% of atypical carcinoids by means of Sanger sequencing [34]. However, lower prevalence rates of mutations in gene belonging to the PI3K/AKT/mTOR pathway have been detected in more recent next-generation sequencing studies, in contrast to neuroendocrine carcinomas [25].

Rare pulmonary neuroendocrine tumors have been described to show a hypermutated profile associated with *POLQ* mutations, a gene involved in DNA double-strand break repair and homologous recombination mechanisms [25, 27]. Moreover, a panel of chromosomal rearrangements, including the *TRIB2-PRKCE* fusion that involves a tyrosine kinase gene, has been recently described and associated with disease recurrence in a cohort of 25 pulmonary carcinoids [29].

Despite the relative abundance of studies, the comparative molecular characterization of typical vs atypical carcinoids is still incomplete. Overall, typical carcinoids have the lowest mutational burden and the lowest mutational rate for each altered gene [35]. The most frequently mutated genes are *EIF1AX*, *ARID1A*, *LRP1B*, and *NF1*. By contrast, atypical carcinoids show a higher rate of mutations for each altered gene and a similar but not identical set of mutations. In fact, although mutation rates of *EIF1AX* and *ARID1A* are in line with those of typical carcinoids, a higher prevalence of *MEN1* mutations is observed in atypical carcinoids as compared with typical carcinoids, reaching 25%. Other genes which are significantly mutated in atypical as compared to typical carcinoids include *SMARCA4*, *ATP1A2*, and *SPHKAP*, all with a mutation frequency exceeding 10% [35]. Interestingly, mutations in genes implicated in chromatin remodeling and histone modification are exclusive (such as *EIF1AX*) or generally more frequent in carcinoids as compared with large-cell and small-cell neuroendocrine carcinomas. It is worth to notice that mutations of other genes involved in chromatin remodeling such as *DAXX or ATRX* are not detected in pulmonary carcinoids, in contrast to other neuroendocrine tumors of foregut origin (i.e., those in the pancreas) [36]. However, in an immunohistochemical study of over 100 cases, it has been shown that loss of ATRX protein is present in 20% of pulmonary carcinoids, with a significant association with atypical carcinoids and shorter disease-specific survival [37].

Molecular alterations that are typical of non-small cell lung cancer, including those that are targets for biologic therapy, are infrequent in pulmonary neuroendocrine tumors. *ALK* or *NTRK* fusions are very rarely detected in pulmonary carcinoids, with only single cases reported [38, 39], including cases with well-differentiated morphology but high proliferative rate [40].

Finally, several molecular studies, in parallel to morphology as discussed above, suggest that a subgroup of carcinoid tumors (mainly atypical carcinoid tumors) and large-cell neuroendocrine carcinomas display a heterogeneity with some overlap between histological types, and that a molecular classification may allow a better prognostic and predictive stratification. For instance, a recent study aimed at the integrative analysis of genome, transcriptome, and methylome data showed that integrated molecular profiles depict specific survival outcomes in patients with carcinoid tumors of atypical morphology, segregating patients with good typical carcinoid-like survival and patients with worse clinical outcome similar to large-cell neuroendocrine carcinomas [27]. In this study, a specific subgroup of neuroendocrine tumors, termed supra-carcinoids, was identified with morphological features of carcinoid tumors but with molecular characteristics similar to large-cell neuroendocrine carcinomas. These tumors were characterized by *MKI67* gene expression levels higher than seen in other carcinoid molecular subtypes, high estimated levels of neutrophil infiltration, altered pathways related to chemotaxis and degranulation of neutrophil, high levels of expression of immune checkpoint receptors and ligands (such as PDL1 and CTLA4), and upregulation of other immunosuppressive genes (including HLA-G and interferon gamma). Moreover, a few small studies were specifically aimed at the molecular characterization of highly proliferative lung carcinoids. In a series of cases at advanced stage of disease, no alterations in *RB1* or *TP53* were detected, whereas mutations on chromatin-modifier genes (*MEN1, ARID1A, ARID1B*, and *KDM5C*) were present in more than 50% of cases [41]. In another study [42], highly proliferative pulmonary neuroendocrine tumors displayed molecular alterations in tumor suppressor genes belonging to pathways commonly altered in both carcinoids and neuroendocrine carcinomas of the lung, including chromatin remodeling, DNA repair, and cell cycle. Moreover, based on data in cases with spatial and/or temporal heterogeneity, this study proposes an evolutionary model from clones of lower aggressivity through the accumulation of “neuroendocrine carcinoma-like” genetic alterations, such as *TP53*/*RB1* alterations.

### Gene Expression Profiling of Pulmonary Neuroendocrine Tumors (Carcinoid Tumors)

Gene expression profiling in pulmonary carcinoids has been assessed mainly in two different settings. The first was tailored to identify gene signatures specific to carcinoid histotypes. A study by Toffalorio et al. [43] identified 273 genes to be upregulated in the atypical vs typical histotype, and among those GC (encoding for a vitamin D-binding protein) and CEACAM1 (encoding for a carcinoembryonic antigen family member) were validated by quantitative PCR and immunohistochemistry and proposed as potent diagnostic markers.

In another setting, gene expression profiling analysis was applied to identify signatures associated with specific biological and clinical behavior. In pulmonary neuroendocrine tumors with poor prognosis, a set of genes was found to be upregulated, including the proto-oncogene *RET* and other genes involved in cell cycle control, such as *ASPM*, *BIRC5*, *BUB1*, *CEP55*, *FANCA*, and others, whereas *OTP*, *PCK1*, *ASB4*, *FOLRI1*, *CD44*, and others were downregulated [44]. Interestingly, *BIRC5*, *BUB1*, *CD44*, *IL20RA*, *KLK12*, and *OTP* were independent predictors of patient outcome. Larger and independent series confirmed the negative prognostic value of the loss of *OTP* and *CD44*, proposing them as relevant clinical biomarkers of aggressiveness [45–47].

In line with genomic data, recent transcriptional studies supported the existence of a grey zone between atypical carcinoid tumors and large-cell neuroendocrine carcinomas. In one study, atypical carcinoid tumors and large-cell neuroendocrine carcinomas have been clustered into three groups. Two groups were enriched for atypical carcinoid tumors or large-cell neuroendocrine carcinomas, respectively, and the genomic findings and outcome of the patients were as would be expected for the respective histotype. A third group was composed of mixed histologies, intermediate molecular features, and a survival similar to atypical carcinoid-type cluster [48]. In another study, neuroendocrine tumors or large-cell neuroendocrine carcinomas were analyzed by RNA sequencing and data matched with clinical and pathological features [49]. The study included four samples classified as borderline neuroendocrine tumor because of the presence of well-differentiated neuroendocrine morphology and increased Ki-67 or mitotic rates. Clustering analysis revealed two distinct molecular groups characterized by low or high proliferation, the former including seven carcinoids and three borderline tumors, and the latter including seven large-cell neuroendocrine carcinomas and one borderline tumor.

### Epigenetic Molecular Mechanisms in Pulmonary Neuroendocrine Tumors (Carcinoid Tumors)

In line with the overall low mutation rates and the few recurrently mutated genes, neuroendocrine tumors are characterized by chromatin remodeling and epigenetic changes, which seem to represent the most relevant molecular mechanisms underlying the pathogenesis of these tumors. Although most of the available data have been obtained in pancreatic and intestinal neuroendocrine tumors, DNA methylation, histone modification, and miRNA expression have also been described in pulmonary carcinoids.

In detail, promoter hypermethylation of *RAS*-association domain family 1 (*RASSF1*) gene has been reported to be a frequent event in typical and atypical carcinoids, with a significant association between higher degrees of promoter methylation and higher tumor grade [50]. Intriguingly, in the same study, a non-linear correlation between mRNA and protein levels was observed, suggesting that post-transcriptional events may regulate the function of *RASSF1* gene [50].

Another gene showing promoter hypermethylation in pulmonary carcinoids is *CDKN2B*, coding for a cyclin-dependent kinase inhibitor p15Ink4b which is functionally similar to the tumor-suppressor gene *CDKN2A* (encoding for the p16INK4a protein), but less well characterized in its tumorigenic potential. It has been shown that the expression of the p15INK4b gene product (p15) was significantly lower in lung neuroendocrine neoplasms, especially in neuroendocrine tumors, as well as in the adjacent normal lung, compared with non-neoplastic control lungs. This finding was at least partially associated with aberrant methylation at the 5′-region of the gene [51]. However, the real role of *CDKN2B* gene promoter hypermethylation in the pathogenesis of pulmonary carcinoids is still a matter of debate [52].

Methylation levels of the *MCAM* gene (a protean modulator of a number of cellular functions, including cell motility and matrix invasion) were demonstrated to be significantly higher in lung carcinoids than in other neuroendocrine neoplasms, supporting its role as a site-specific molecular biomarker [53]. More recently, the *KEAP1* gene silencing by promoter methylation was reported in about 50% of typical and atypical lung carcinoids, which correlated with reduced protein expression [54, 55].

The importance of the regulation of DNA methylation in the pathogenesis of pulmonary neuroendocrine tumors is also reflected by the finding of the overexpression in these tumors of enzymes active in DNA methylation. For example, nuclear overexpression of the protein arginine methyltransferase-5 (*PRMT5*), which is a chromatin-modifying enzyme, was reported to be more frequent in pulmonary carcinoids than in neuroendocrine carcinomas, suggesting different epigenetic regulatory mechanisms controlling oncogenesis in carcinoids as compared with high-grade pulmonary neuroendocrine neoplasms [56]. Conversely, tumor grade has been shown to be inversely correlated with expression levels of the histone methyltransferase enhancer of zeste homolog 2 (*EZH2*), being identified in neuroendocrine carcinomas of the lung, but lacking in pulmonary carcinoids [57].

A few systematic studies are available regarding histone modifications in pulmonary carcinoids. The expression of histone H4 acetylation at lysine 16 (H4KA16) and trimethylation at lysine 20 (H4KM20) was analyzed in a series of 32 pulmonary neuroendocrine neoplasms, showing a progressive loss of H4KM20 and H4KA16 along the spectrum of neuroendocrine neoplasms. Namely, typical carcinoids showed no alterations of histone H4, whereas early changes were observed in atypical carcinoids, and widespread loss of H4KM20 and H4KA16 was present in neuroendocrine carcinomas [58].

Concerning non-coding RNAs, the analysis of miRNAs expression profile in pulmonary neuroendocrine tumors demonstrated peculiar features which are different from neuroendocrine carcinomas of the lung and more similar to digestive neuroendocrine tumors [59–62]. Interestingly, typical and atypical carcinoids have been reported to differentially express specific miRNA subsets and, in addition, distinct miRNA expression profiles have been described in metastatic cases [59, 60].

Long non-coding RNA profiling in pulmonary neuroendocrine neoplams has only been explored in a single study. In that study, the expression of HOX transcript antisense RNA (*HOTAIR*), maternally expressed 3 (*MEG3*), and prostate cancer antigen 3 (*PCA3*) has been found to be significantly lower in pulmonary carcinoids as compared with neuroendocrine carcinomas. The functional significance of this observation still needs to be explored [63].

Overall, our current knowledge of epigenetic changes involved in the molecular pathogenesis of pulmonary neuroendocrine tumors not only represents additional and complementary information to better understand tumor development and progression but also provides useful diagnostic tools in the distinction between carcinoids and neuroendocrine carcinomas. In addition, several epigenetic mechanisms are potential therapeutic targets in view of a tailored target therapy.

### Molecular Background of Thymic Neuroendocrine Tumors (Carcinoid Tumors)

Using low-coverage whole-genome sequencing and copy number instability (CNI) scoring, Dinter et al. showed that the average CNI ranges from 5.25 in typical carcinoids to 18.3 in atypical carcinoids and 44.4 in neuroendocrine carcinomas [19]. However, outliers were observed specifically in cases of atypical carcinoids and large-cell neuroendocrine carcinomas. Overall, based on CNI, the authors were able to identify three clusters in which all thymic neuroendocrine neoplasms fell. CNI_low_ (CNI < 9), CNI_int_ (CNI 9- < 30), and CNI_high_ (CNI ≥ 30). CNI_low_ and CNI_int_ comprised typical and atypical carcinoid tumors and 44% of large-cell neuroendocrine carcinomas. CNI_high_ contained all small-cell carcinomas, 56% of large-cell neuroendocrine carcinomas, and 10% of atypical carcinoids. Interestingly, the CNI clusters correlated with survival. However, the CNI clusters could not be predicted by any morphologic feature, mitotic count, or Ki-67 labeling index. Therefore, the authors proposed a “morphomolecular grading system” for thymic neuroendocrine neoplasms to better predict the prognosis of these tumors (Table 1). The authors also showed that at least in a subset of neuroendocrine neoplasms of the thymus, there might be progression from low- to higher-grade histologies and found some additional focal chromosomal gains and losses in metastatic disease.

**Table 1:** Modified proposal for a “morphomolecular grading system” of thymic neuroendocrine tumors ( Modified from Dinter et al. [19])

Thymic NET grade^a^	Morphology WHO subtype	Mitotic Index (mitoses per 2mm^2^)	Ki-67 (%)	CNI score	Immunohistochemistry
G1	Carcinoids	< 10	< 9	< 9	Chromogranin pos^c^ EZH2 neg^d,e^
G2	Carcinoids^b^ Large-cell NEC	10–29	9–47	9–29	Chromogranin pos EZH2 neg
G3	Large-cell NEC Small-cell NEC	≥ 30	≥ 48	≥ 30	Chromogranin pos/neg EZH2 pos

*NEC* neuroendocrine carcinoma

^a^Proposed morphomolecular thymic neuroendocrine tumor grade

^b^Carcinoid morphology subtypes also include typical and atypical carcinoids as well as NET G3; the latter is similar to grade-discordant gastroenteropancreatic tract neuroendocrine tumors that are characterized by a well-differentiated (carcinoid) morphology and mitotic count like G2 tumors but increased Ki-67 index (> 20%) equivalent to G3 tumors [107]

^c^Pos, expressed

^d^Neg, not expressed

^e^EZH2 neg defined as < 25% of tumor cell staining

Interestingly, in contrast to pulmonary carcinoids, 11q13 deletions were not identified in thymic neuroendocrine tumors [64, 65]. Gross chromosomal imbalances, as detected by means of comparative genomic hybridization (CGH), occur in 31–88% of cases [65, 66]. Similar to the study by Dinter et al. [19], an earlier study by Stroebel et al. [66] using CGH has shown an incremental increase of genetic alterations from typical to atypical carcinoid to neuroendocrine carcinomas with mean numbers of aberrations per tumor of 0.8 in typical carcinoids, 1.1 in atypical carcinoids, 4.7 in large cell neuroendocrine carcinomas, and 15.5 in small-cell carcinomas. The most common findings of CGH studies of thymic neuroendocrine tumors are summarized in Table 2. Frequent overlapping genetic alterations were found in both neuroendocrine tumors and neuroendocrine carcinomas [66]. In that study, a cut-off of 2.5 chromosomal imbalances correlated with a higher rate of recurrence and death rate.

**Table 2:** Results of comparative genomic hybridization studies in thymic neuroendocrine tumors (carcinoids) [19, 62]

Subtype of thymic neuroendocrine tumor	Molecular alteration
Typical carcinoid tumors	Large copy number alterations including Gains on chromosomes 1q, 5, 6q, 7q, 8q, 10, 11q, 12q, 13q, 18q, 20, 21q, 22q Losses on chromosomes 1, 2p, 4p, 6q, 8, 10p, 10q, 11p, 13q, 15q, 17p, 18p, 22q Deletions in gene loci on chromosomes 1p, 3q, 8p, 11q, 17p
Atypical carcinoid tumors	Large copy number alterations including Gains on chromosomes 1q, 7p, 7q, 10, 12q, 20q, 21, 22, Xp Losses on chromosomes 1p, 2p, 3p, 4p, 4q, 6q, 10p, 11p, 13q, 17p Deletions in gene loci on chromosomes 1p, 3p, 3q, 5p, 6p, 6q, 9p, 10q, 13q, 17p Amplifications in gene loci on chromosomes 8q, 14q

Recent studies have suggested that mRNA transcript analysis in blood can identify thymic neuroendocrine tumors [5]. Three non-secretory atypical carcinoid tumors (i.e., no production of serotonin or ACTH) with low chromogranin A levels were found to have detectable expression of genes that have been linked to neuroendocrine tumor pathobiology in the circulating blood using the NETest [5, 67]. However, these findings need to be validated in larger cohorts and their clinical utility in identifying progressive tumors, confirming complete tumor resection, and predicting treatment response has to be established.

Mutational burden is low in thymic neuroendocrine tumors with < 1 chromosomal alteration per tumor which occurs in less than a third of patients [66]. Approximately 25% of patients with thymic carcinoid tumors harbor a *MEN1* germline mutation [68] and 8% of MEN1 patients develop thymic neuroendocrine tumors [69]. However, loss of heterozygosity of the *MEN1* locus on chromosome 11q13 has not been described in thymic carcinoids [65, 70]. One sporadic thymic carcinoid was reported to harbor a nonsense mutation in *MEN1* (Q393X) [71].

Evidence suggests that *PAK3* (p21-activated kinase 3) might play a role in the progression of ACTH-producing thymic carcinoids [72]. Complementary DNA profiling of 7 ACTH-producing thymic carcinoids identified 5 adhesion-related genes to be downregulated and 5 to be upregulated including a remarkable overexpression of *PAK3* in all tumors. Its upstream regulator, *RAC1*, was also overexpressed. The authors showed that *PAK3* overexpression was associated with enhanced cell migration and invasion. Interestingly, *PAK3* expression levels were higher in tumors with progressive disease than tumors without. ACTH-secreting tumors were also found to express elevated beta-catenin and decreased *NOTCH2* levels, genes involved in the Wnt signaling pathway. Not surprisingly, the neuropeptide signaling pathway was also upregulated, but it was thought that this might have been a consequence of the tumor.

In thymic neuroendocrine tumors, there are currently no potential prognostic biomarkers or targetable molecular alterations known [73]. Furthermore, circulating tumor DNA has not been studied. Therefore, future studies concerning prognostic biomarkers that might better predict the behavior of an individual tumor and targetable molecular aberrations or neoplastic cell proteins are important for the management and treatment of patients with thymic neuroendocrine tumors.

### Germline Variants in Lung and Thymic Neuroendocrine Tumors

Most pulmonary and thymic neuroendocrine tumors (carcinoid tumors) are associated with sporadic disease; thus, only a small fraction is currently linked to germline susceptibility [74]. The age of the patient at time of diagnosis, personal, and/or family history for inherited endocrine tumor syndromes or tumors that may be seen in association with various neuroendocrine neoplasms, cytomorphological findings, and molecular immunohistochemical biomarkers are all important parameters that might prompt diagnosticians to search for a potential underlying germline disease in neuroendocrine neoplasia [74]. Similar to other anatomic sites, the accurate distinction of paragangliomas (non-epithelial neuroendocrine neoplasms of paraganglia) from their epithelial counterparts is of significance since primary pulmonary and mediastinal paragangliomas are more frequently associated with germline alterations [75] compared with thoracic (pulmonary and thymic) neuroendocrine tumors. For this reason, diagnostic immunohistochemical biomarkers of paraganglia (e.g., tyrosine hydroxylase, dopamine beta-hydroxylase, and GATA3) should be applied to all keratin-negative neuroendocrine neoplasms [75–77].

The true incidence of germline susceptibility is currently difficult to determine since clinical studies focusing on germline variants in pulmonary and thymic neuroendocrine tumors are scant. This is partially due to the lack of appreciation of germline disease in apparently sporadic-appearing thoracic neuroendocrine neoplasms given the discovery of germline DNA repair defects in non-syndromic thoracic neuroendocrine tumors [78]. Ideally, a whole-exome sequencing of germline DNA from all young patients with thoracic neuroendocrine neoplasm and/or any patient with pulmonary neuroendocrine disease either in the form of pulmonary neuroendocrine cell hyperplasia-to-neoplasia sequence or multifocal pulmonary neuroendocrine disease (e.g., solitary pulmonary neuroendocrine tumor in a background of widespread pulmonary neuroendocrine cell hyperplasia, or pulmonary neuroendocrine microtumors—also known as carcinoid tumorlets, or multifocal pulmonary neuroendocrine tumors) should be integrated into clinical management guidelines.

**Fig. 4:**
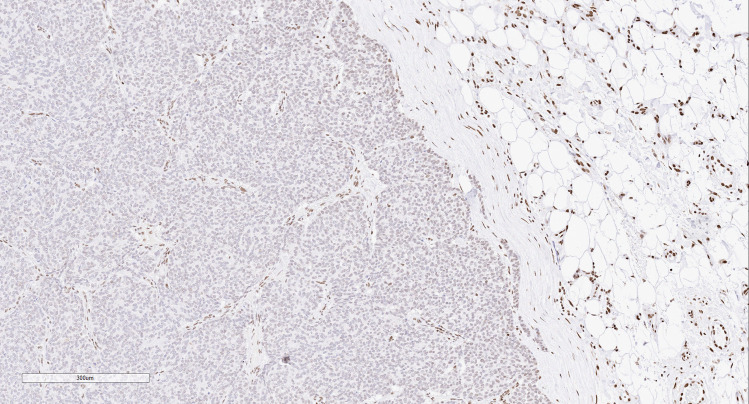
MEN1 patient with a thymic neuroendocrine tumor that shows diffuse loss of nuclear menin expression, while the non-tumorous elements (internal control) retain nuclear staining for menin

Among syndromic manifestations, pathogenic *MEN1* variants that cause MEN1 syndrome account for the most well-defined inherited syndrome that can feature thoracic neuroendocrine tumors [79–81]. Among patients with MEN1 syndrome, thymic neuroendocrine neoplasms, which are considered an important source of morbidity, were reported to occur in 3.7% of MEN1 patients in a recent meta-analysis [79, 80, 82], whereas pulmonary neuroendocrine neoplasms have been noted in approximately 2–13% of MEN1 patients [79, 81, 83–87]. The histopathology of MEN1-related lung disease includes more common multifocal neuroendocrine proliferations such as pulmonary neuroendocrine micro-tumors (carcinoid tumorlets) that can be seen in association with pulmonary neuroendocrine cell hyperplasia [81]. The pulmonary neuroendocrine cell hyperplasia-to-neuroendocrine neoplasia progression sequence in MEN1 syndrome can also be screened using molecular immunohistochemistry. For instance, diffuse loss of nuclear menin (protein encoded by *MEN1*) immunostaining in multifocal pulmonary neuroendocrine (micro)tumors or thymic neuroendocrine tumors should alert diagnosticians to the possibility of MEN1 syndrome even in the absence of a family history [88] (Fig. 4). The diagnosis is further justified by demonstrating *MEN1* defect in the germline DNA (e.g., blood).

A small fraction of patients with a negative germline *MEN1* testing but with MEN1-like clinical and pathological features present with MEN4 syndrome. Affected patients frequently harbor germline pathogenic *CDKN1B* defects [79, 89]. The true incidence of MEN4 syndrome in the context of well-differentiated thoracic neuroendocrine tumors is currently unknown. However, diffuse loss of nuclear p27 (protein encoded by *CDKN1B*) expression in the appropriate clinical and morphological setting can help rationalize genetic screening to rule out germline *CDKN1B*-related thoracic neuroendocrine neoplasia [88].

The identification of pathogenic germline *RAD51AC* defects in a thymic neuroendocrine tumor [78], as well as a germline *APC* variant harboring breast carcinoma patient with a pulmonary neuroendocrine tumor [90] and a germline *MSH2* variant of unknown significance in another breast carcinoma patient with well-differentiated pulmonary neuroendocrine tumor [90], expands the spectrum of non-syndromic manifestations of germline disease in well-differentiated thoracic neuroendocrine neoplasms.

Germline *NKX2.1* mutations (encoding TTF1) have been reported in patients and their adult family members with familial pulmonary neuroendocrine cell hyperplasia of infancy [91, 92]. Although the pulmonary involvement often shows significant improvement with age, rare examples of adult patients with familial neuroendocrine cell hyperplasia of infancy showed overlapping imaging findings with diffuse idiopathic pulmonary neuroendocrine cell hyperplasia (DIPNECH) [92]. Interestingly, an adult Japanese patient with Notch3-related cerebral autosomal dominant arteriopathy with subcortical infarcts and leucoencephalopathy developed DIPNECH as well as other non-neuroendocrine neoplasms including prostatic adenocarcinoma, renal cell carcinoma, and adenomatoid tumor (epididymis) [93]. In addition, the occurrence of DIPNECH in the setting of multiple epithelial neoplasms of various organs including papillary thyroid carcinoma, breast carcinoma, and carotid body paraganglioma in a patient with pathogenic *PALB2* is also of great interest [94].

While germline variants in patients with DIPNECH require further studies, the documentation of a common breast carcinoma history and associated germline defects in patients with pulmonary neuroendocrine disease is an interesting pathogenetic association as seen in other neuroendocrine neoplasms [90, 94]. More importantly, these findings suggest that the spectrum of germline disease in thoracic neuroendocrine neoplasia is likely underestimated. Therefore, implementation of routine genetic screening protocols for germline screening is indicated to better understand its incidence and underlying molecular cellular mechanisms.

## Implications for Therapy

Therapeutic strategies and lines of treatment in pulmonary and thymic neuroendocrine tumors in inoperable or progressive cases are poorly defined in current clinical guidelines [95]. In these patients, first-line treatment includes somatostatin analogs alone or in combination with other therapies, or cytotoxic chemotherapy. These same treatments are also the most adopted as second-line therapy, together with mTOR inhibitors and other regimens in a wide range of schemes and sequences that are heterogeneous even within single institutions [96]. Molecular predictive biomarkers in the clinical setting are mostly missing. Specific profiles of expression of somatostatin receptors (mainly loss of expression of somatostatin receptors type 1 and 2 and increased expression of somatostatin receptors type 3 and 4) have been associated with the presence of aggressive features and shorter survival in untreated patients [97]. However, although somatostatin analogs proved to be effective treatments in aggressive pulmonary carcinoids [98], somatostatin receptor expression has no role in predicting the response to somatostatin analogs in thoracic well-differentiated neuroendocrine neoplasms. Alkylating agents are the mainstay for chemotherapy, and deficiency of O6-methylguanine-methyltransferase (MGMT) enzyme has been associated with a better response to these agents [99]. However, in lung well-differentiated neuroendocrine tumors, despite MGMT being shown to be deficient in approximately 50% of cases [100], no data are available on a predictive role of MGMT status in chemotherapy-treated patients. Although some lines of therapy are based on the biological evidence of the disruption of specific pathways in thoracic neuroendocrine tumors, no clinically relevant molecular biomarker is used to select or predict patient’s response to a given treatment. Several molecular alterations in thoracic neuroendocrine tumors interfere with cell-cycle regulation, chromatin remodeling, apoptosis, intracellular cascades, and cell–cell interactions. However, molecular alterations in thoracic neuroendocrine tumors that might be considered as targets for treatment are very scarce, and treatment of pulmonary and thymic carcinoids on a personalized basis is still an unmet goal, despite a list of hypothetical biomarkers [101]. Among those, rovalpituzumab tesirine, which targets the inhibitory Notch ligand Delta-like protein 3 (DLL3), has been shown to be a promising therapeutic agent in pulmonary neuroendocrine carcinomas. Although to a lower extent as compared with small cell lung carcinoma, high DLL3 expression was observed in 37% of atypical carcinoids and 32.8% of typical carcinoids [102]. However, the impact of DLL3-targeted therapy in aggressive pulmonary neuroendocrine tumors is unexplored, so far. Chromatin-modifying genes could also be implicated in the treatment of thoracic well-differentiated neuroendocrine neoplasms. As an explicatory model, preliminary literature data showed that *ARID1A* alterations increase tumor cell sensitivity to agents targeting the ATR protein, EZH2 or the PI3K pathway [103–105]. Moreover, *ARID1A* alterations interfere with mismatch repair pathway and immunoregulatory mechanisms. In fact, ARID1A has been shown to recruit MSH2 to chromatin during DNA replication, and ARID1A inactivation therefore compromises mismatch repair mechanisms. Moreover, in vivo* ARID1A*-deficient ovarian cancer models exhibit an increased number of tumor-infiltrating lymphocytes, a higher tumor mutation load, and elevated PD-L1 levels [106]. The supracarcinoid cluster described in the study by Alcala et al. [27] also showed peculiar immune-to-tumor response profiles that suggest a potential role for immunotherapy in aggressive carcinoids of the lung. However, all molecular data described in thoracic neuroendocrine tumors so far lack a direct impact in terms of therapy and still deserve both functional and clinical validation as biomarkers to be integrated to pathological characterization.
